# Doctoral physical therapy students’ increased confidence following exploration of active video gaming systems in a problem-based learning curriculum in the United States: a pre- and post-intervention study

**DOI:** 10.3352/jeehp.2022.19.7

**Published:** 2022-04-26

**Authors:** Michelle Elizabeth Wormley, Wendy Romney, Diana Veneri, Andrea Oberlander

**Affiliations:** Department of Physical Therapy & Human Movement Science, Sacred Heart University, Fairfield, CT, USA; Hallym University, Korea

**Keywords:** Delivery of health care, Exergaming, Physical therapy modalities, Problem-based learning, United States

## Abstract

**Purpose:**

Active video gaming (AVG) is used in physical therapy (PT) to treat individuals with a variety of diagnoses across the lifespan. The literature supports improvements in balance, cardiovascular endurance, and motor control; however, evidence is lacking regarding the implementation of AVG in PT education. This study investigated doctoral physical therapy (DPT) students’ confidence following active exploration of AVG systems as a PT intervention in the United States.

**Methods:**

This pretest-posttest study included 60 DPT students in 2017 (cohort 1) and 55 students in 2018 (cohort 2) enrolled in a problem-based learning curriculum. AVG systems were embedded into patient cases and 2 interactive laboratory classes across 2 consecutive semesters (April–December 2017 and April–December 2018). Participants completed a 31-question survey before the intervention and 8 months later. Students’ confidence was rated for general use, game selection, plan of care, set-up, documentation, setting, and demographics. Descriptive statistics and the Wilcoxon signed-rank test were used to compare differences in confidence pre- and post-intervention.

**Results:**

Both cohorts showed increased confidence at the post-test, with median (interquartile range) scores as follows: cohort 1: pre-test, 57.1 (44.3–63.5); post-test, 79.1 (73.1–85.4); and cohort 2: pre-test, 61.4 (48.0–70.7); post-test, 89.3 (80.0–93.2). Cohort 2 was significantly more confident at baseline than cohort 1 (P<0.05). In cohort 1, students’ data were paired and confidence levels significantly increased in all domains: use, Z=-6.2 (P<0.01); selection, Z=-5.9 (P<0.01); plan of care, Z=-6.0 (P<0.01); set-up, Z=-5.5 (P<0.01); documentation, Z=-6.0 (P<0.01); setting, Z=-6.3 (P<0.01); and total score, Z=-6.4 (P<0.01).

**Conclusion:**

Structured, active experiences with AVG resulted in a significant increase in students’ confidence. As technology advances in healthcare delivery, it is essential to expose students to these technologies in the classroom.

## Introduction

### Background/rationale

Over the last 2 decades, physical therapists have incorporated physically interactive gaming systems (e.g., Nintendo Wii, Xbox Kinect, PlayStation) into patient treatment sessions to address a variety of impairments in capacities such as strength, balance, coordination, motor control, and endurance across the lifespan [1]. Active video gaming (AVG) or screen-based activities are those that “require increased physical activity to play the game compared to conventional sedentary, or passive video games” [2]. These off-the-shelf systems are used by physical therapists to offer patients a means to stay motivated, improve compliance, address targeted impairments, and improve function and outcome measures during rehabilitation [3]. Given the implementation of AVG in physical therapy practice, students enrolled in a doctorate of physical therapy (DPT) curriculum should have the opportunity to experience video gaming as a patient treatment intervention.

In this study, we used an active learning approach to expose DPT students to AVG at multiple time points in a problem-based learning (PBL) curriculum to investigate their perceived confidence level with AVG systems. A gap in the literature exists regarding student exposure to AVG in PT curricula. The use of AVG systems has been well documented across the lifespan. Pediatric diagnoses treated utilizing AVG included burns, autism, and acquired brain injury [4]. In adolescent patient populations, AVG has been used to treat patients with intellectual disabilities, to rehabilitate patients after anterior cruciate ligament repair, and to treat patients with metacarpal fracture [5,6]. In adults with stroke, a variety of outcomes have been measured including balance and gait, upper and lower extremity function, activities of daily living, sensorimotor function, and quality of life [7].

A meta-analysis performed by Taylor et al. [8] concluded that AVG can improve measures of mobility and balance in older adults when used either on its own or as part of an exercise program. AVG has also been used with frail elderly individuals to address the feasibility of an intervention with implications for reducing fall risk [9]. Active participation is a preferred learning style of PT students and therefore aligns well with this study’s planned video gaming experience [10]. Experiential learning (“learning by doing”) has been shown to improve self-confidence, communication, and attitudes and beliefs toward specific patient populations. Experiential learning is consistent with the PBL approach. Becoming an active learner is an underpinning of the PBL curriculum.

### Objectives

Considering the evidence-based, widespread clinical application of AVG with a variety of patients across the lifespan, as well as the positive outcomes using active learning approaches, we aimed to fill a gap in the literature by implementing AVG via an active learning approach with DPT students and evaluate their perceived confidence. Therefore, the purpose of this project was to investigate DPT students’ perceived confidence with the use of AVG before and after active laboratory experiences using AVG. The authors hypothesized that the implementation of AVG systems within a PBL curriculum would increase DPT students’ perceived confidence with general use, game selection, plan of care, set-up, documentation, and the practice setting.

## Methods

### Ethics statement

Institutional Review Board approval was obtained through Sacred Heart University (IRB #171201E). Informed consent was implied by completing the surveys.

### Study design

This quasi-experimental study had a pre- and post-test design. It is described according to the STROBE (Strengthening the Reporting of Observational Studies in Epidemiology) statement.

### Setting

The PBL curriculum at Sacred Heart University consists of 5 academic semesters and 38 weeks of clinical education. Semesters 2 and 3 of the DPT program focus on examination and treatment strategies for patients with neurological dysfunction and medically complex issues. A variety of patient populations across the lifespan are covered, including patients with stroke, traumatic brain injury, Parkinson’s disease, multiple sclerosis, cerebral palsy, amputation, spinal cord injury, burns, and cardiopulmonary disease.

The details and timeline (April 2017 to December 2018) for this study are summarized in Table 1. In semester 2, the focus was on including AVG systems as a mode of therapeutic exercise and balance retraining for patients with neurological diagnoses. The curriculum included the use of patient case-based tutorials throughout each semester to integrate content across basic science, clinical science, and professional development courses. Five to seven students and a tutorial leader met for tutorial sessions twice weekly for 3 hours each session. A prompt to explore AVG within the curriculum was added to a tutorial case for a patient who sustained a concussion (Supplement 1). This particular case was chosen to introduce the concepts of AVG due to the patient’s age, interest in sports/adventure activities, and resulting neurological impairments suffered from the concussion including visual-perceptual, dual-tasking, and visual-motor difficulties.

**Fig. 1.**
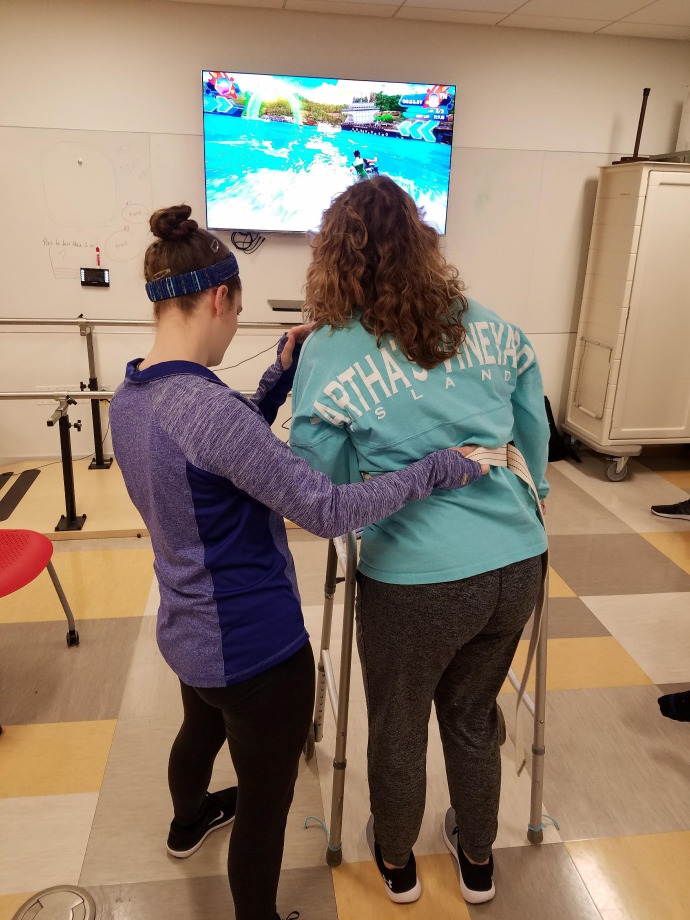
Doctoral physical therapy students participating in an active video gaming lab experience.

An outline was created for lab activity #1 (Supplement 2), based on the work of Levac et al. [11]. This was done to provide operational definitions for game characteristics and provide exposure to different games, activities, and choice of patient position based on functional ability. The cases used in the lab included the previously-mentioned tutorial case, and 2 prior tutorial cases the students were familiar with: an individual with Parkinson’s disease and an individual post-stroke to represent a variety of diagnoses and functional levels. During the labs, students rotated to 3 stations in small groups for 45 minutes each with a faculty facilitator. Students acted as the patient to appreciate game operation, physical requirements, and level of difficulty. They also approached each station as the therapist to practice guarding skills, cueing, monitoring, modifying and considering exercise prescription (Fig. 1, Supplement 3). Students were asked to reflect on 7 guiding questions at each station and at the end of the lab during a full class debrief (Supplement 2).

Upon completion of the semester, students participated in an 8-week summer clinical placement. Students returned as second-year students to participate in semester 3 of the curriculum. In this semester, students were re-introduced to AVG in a tutorial case related to a medically complex diagnosis with subsequent lab activities including this case and past tutorial cases. The outline and lab rotation for lab activity #2 (Supplement 4) were similar to lab activity #1.

### Participants

All first-year students enrolled in the DPT program in April 2017 (cohort 1) and April 2018 (cohort 2) were invited to participate (n=122).

### Variables

The dependent variable included changes in students’ perceived confidence for general use of gaming, game selection, plan of care, set-up, documentation, setting, and total score as measured by a confidence survey.

### Data sources/measurement

The baseline confidence survey (Supplement 5) was adapted from Erlich and Russ-Eft [12] and emailed to students via SurveyMonkey (SurveyMonkey, San Mateo, CA, USA) before they participated in lab activity #1. The original tool used principal component analysis (PCA) to identify factors within each section. Cronbach’s α (0.91) was applied to further reduce the number of items for each identified factor while retaining internal consistency reliability. Factorial validity was supported by the interpretability of the PCA results [13]. The 31-item survey included 3 demographic questions and assessed students’ perceived confidence across 6 domains: general use of gaming (5 questions), game selection (7), plan of care (5), set-up (3), documentation (3), and setting (5). The internal consistency reliability of the current survey had a Cronbach’s α of 0.97. Students were instructed to rate their level of confidence in each domain on a 0–100 scale (no confidence=0, 10; limited confidence=20, 30, 40; moderate confidence=50, 60, 70; high confidence=80, 90, 100). The post-test was completed at the end of lab activity #2, 8 months after the pre-test. The intentional gap in the time allowed students to gain experience in their first clinical placement to develop a point of reference and the opportunity for multiple exposures within the curriculum.

### Bias

The authors attempted to minimize biases by including 2 different cohort years of students within the program.

### Study size

This was a sample of convenience based on the number of DPT students enrolled in the program during the study timeline (April 2017 to December 2018). Based on an effect size of 0.52, the sample size required was 44.

### Statistical methods

The data from the surveys were downloaded from SurveyMonkey into an Excel spreadsheet. Descriptive data were included if more than 50% of the surveys were completed. Data were analyzed descriptively by cohort. Medians and interquartile ranges (IQRs) were calculated by each domain as the data were skewed. Data were entered into IBM SPSS ver. 26.0 (IBM Corp., Armonk, NY, USA) to determine baseline differences in cohorts using the Mann-Whitney U test. The Wilcoxon signed-rank test compared the differences in confidence pre- and post-intervention for cohort 1, as most of the data were not normally distributed, as evidenced by the Shapiro-Wilk test (P<0.05). The effect size was calculated for the domain scores and total score using r=Z/square root of N. Data from cohort 2 could not be matched and therefore the Wilcoxon signed-rank test was not completed.

## Results

### Participants

Two consecutive cohorts of students enrolled in semesters 2 and 3 of a DPT curriculum participated (cohort 1: April 2017–December 2017; n=60; and cohort 2: April 2018–December 2018; n=55). The majority of the participants were female with undergraduate degrees in exercise science (Table 2).

### Main results

Students’ responses to the pre- and post-intervention surveys are available in Dataset 1 and Dataset 2. The pre- and post-intervention medians and IQRs for the cohorts are presented in Table 3. Both cohorts showed increased confidence, with median (IQR) scores as follows: cohort 1 total score, pre-intervention: 57.1 (44.3–63.5), post-intervention: 79.1 (73.1–85.4), and cohort 2: pre-intervention: 61.4 (48.0–70.7), post-intervention: 89.3 (80.0–93.2). Cohort 2 was significantly more confident than cohort 1 at baseline in 4 of the 5 domains including creating a plan of care (mean rank for cohort 1=49.39, mean rank for cohort 2= 64.47, U=1,170.0, P=0.014), video game set-up (mean rank for cohort 1=48.04, mean rank for cohort 2=64.96, U=1,094.9, P=0.006), documentation (mean rank for cohort 1=49.12, mean rank for cohort 2=63.88, U=1,154.5, P=0.016) and setting (mean rank for cohort 1=46.44, mean rank for cohort 2=66.56, U=1,004.5, P=0.001) (Dataset 3). For cohort 1, students’ confidence levels increased significantly compared to baseline testing in all domains: use, Z=-6.2 (P<0.01); selection, Z=-5.9 (P<0.01); plan of care, Z=-6.0 (P<0.01); set-up, Z=-5.5 (P<0.01); documentation, Z=-6.0 (P<0.01); setting, Z=-6.3 (P<0.01); and total score, Z=-6.4 (P<0.01). The effect size was large for the 5 domains (range, r=0.52–0.62 and for the total score, r=-0.62). Using the G*Power calculator (Heinrich-Heine-Universität Düsseldorf, Düsseldorf, Germany; http://www.gpower.hhu.de/), an effect size of 0.52, α of 0.05, and power of 95%, the total sample size required for cohort 1 was 44, which was achieved.

## Discussion

### Key results

This quasi-experimental study evaluated DPT students’ perceived confidence related to the use of AVG systems within a PBL curriculum. Student confidence levels increased significantly in domains including general use of gaming, video game set-up, plan of care, documentation, and setting, as well as the total score.

### Interpretation

We attribute the improved confidence to the clinical utility of the AVG systems chosen, as well as the active learning that occurred during the laboratory activities. This active versus passive learning process aligns with the underpinnings of the PBL curriculum. Embedding the case into the PBL tutorial for self-exploration, research, and discussion before lab and an intended gap between AVG experiences across the curriculum, allowed for time to reflect on the skills learned, an essential aspect of the experiential learning process.

#### AVG system selection

When determining the AVG system that would be most feasible for implementing in the DPT curriculum, it was important to consider clinical utility, patient-friendly capability (no training or background required), and cost-effectiveness. The Nintendo Wii, Nintendo Wii Switch, and Xbox Kinect are video game consoles available for purchase at a reasonable price for clinics and patients to transition their AVG clinical plan of care to a home exercise program. Because these systems are intended for home use, the equipment does not require extensive physical space. We also wanted to be mindful of the cost, interest, ease of use, and availability across the lifespan, and therefore we selected an older gaming system (the Nintendo Wii) in addition to more current systems (Xbox One Kinect and Nintendo Switch) to provide feasible options for clinic and home use. The literature suggests that an optimal game design for rehabilitation includes increasing patient engagement by providing rewards, optimal challenges, feedback, choices/interactivity, clear instructions, and socialization [3]. The selection of games in this study offered a variety of gross and fine motor requirements, cognitive levels, and interests including slalom skiing, table tilt, and yoga (Nintendo Wii Fit with balance board), soccer, rock climbing, wave runner, and Fruit Ninja (Xbox Kinect), and Just Dance (Nintendo Switch).

#### Active Learning and PBL

Active learning is consistent with the underpinnings of the PBL approach, as active learning enhances retention and recall, activation of prior knowledge facilitates the processing of new information, elaboration of knowledge at the time of learning enhances retrieval, matching context facilitates recall, and repeated exposure to PBL sessions improves students’ problem-solving skills by refining their scientific reasoning processes. A qualitative study with DPT students in this PBL program previously reported the experience of transforming into an “active learner” in both the classroom and the clinic environment and suggested that this change was due to the PBL curriculum [14].

The PBL design is in agreement with the scoping review by Stander et al. [10] that learning activities need to be aligned to ensure the learners are given the necessary theoretical backing, and then given enough opportunity to practice and apply the theory. In our study, the concept of AVG was introduced in a tutorial at the end of the semester (tutorials no., 19/24). This was strategically placed at the end of the semester, after students had the opportunity to investigate a variety of clinical diagnoses, develop the ability to critically appraise the literature, and determine interventions appropriate to a set of given exam findings. The students were prompted to research the evidence surrounding the application of AVG in PT practice. At the next tutorial session, students had the opportunity to discuss their findings within tutorial groups to gain foundational knowledge and reflect on discussions prior to attending the lab.

During the lab experience, students actively took part in learning by role-playing the patient and the therapist through the use of case studies. The use of case studies allowed students to have discussions related to clinical reasoning when determining the physical and cognitive requirements for each game. This resulted in the need to adjust their guarding and patient position, make modifications to the equipment such as bandaging the hand used for control for an individual with a spinal cord injury, and determine how/when to take vital signs during these activities to monitor safety and aerobic intensity.

Another critical component of experiential learning is to allow time for reflection [15]. Although informal, students were given time to reflect on knowledge gleaned from the tutorial prior to attending lab, during the faculty-facilitated debriefing at the end of the lab, and between the 2 lab experiences. The 8-month gap between the pre-test (April) and post-test (December) also allowed for students to reflect as they completed their first 8-week clinical experience (in an outpatient or inpatient rehabilitation environment) followed by the majority of the third semester of the DPT curriculum before participating in lab activity #2.

### Limitations

Potential limitations of this study include possible differences in student exposure/experiences with AVG systems and games at baseline, a small sample of DPT students from a single university in the Northeast region of the United States, and the fact that the changes reported were based on student self-perceptions as opposed to actual change in student knowledge. The study lacked randomization as it had a single group quasi-experimental pre-test post-test design.

### Generalizability

The findings from this study support the need to implement active learning strategies to improve DPT self-confidence when using AVG systems as part of a plan of care with a variety of diagnoses across the lifespan, with the ultimate goal of better preparing physical therapists for clinical practice.

### Suggestions

Future research is recommended to develop a tool to evaluate knowledge acquisition and psychomotor performance, determine student perceptions versus behavior, and advance from AVG to implementing virtual reality in the curriculum. Investigating the implementation of AVG and subsequently virtual reality across curricular designs and assessing its application in clinical practice would also assist in determining the effectiveness of this addition.

### Conclusion

Implementation of AVG systems within a PBL curriculum increased DPT students’ perceived confidence in the following domains: general game use, plan of care, set-up documentation, and practice setting, as well as the total score. Considering the increased use of AVG systems in patients with neurological and medically complex diagnoses in clinical practice, the implementation of this skill in the curriculum has the potential to improve student confidence when transitioning into clinical practice.

## Figures and Tables

**Table 1.** Intervention description and timeline

Timeline	Intervention
Month 0: semester 2 (Spring); tutorial case	Students explored literature related to incorporating the use of active video gaming into the physical therapy plan of care.
Week 2: baseline; lab activity #1; 45 minutes in small groups at each station followed by a 30 minute debrief with the class (Supplement 2)	Pre-test survey was emailed and completed prior to lab activity #1
	^a)^Station 1: 73-yr female; s/p left middle cerebral artery stroke
	• Stability: static standing postural control without an assistive device
	• AVG system: Nintendo Wii
	• Game(s): Wii Fit with balance board for slalom skiing, table tilt, yoga
	^b)^Station 2: 82-yr female with Parkinson’s disease
	• Mobility: standing dynamic postural control
	• AVG system: Nintendo Switch
	• Game: Just Dance
	^c)^Station 3: 27-yr male; s/p post-concussion while snowboarding
	• Mobility Plus: high-level postural control activities
	• AVG system: Xbox One Kinect
	• Game(s): soccer, rock climbing, wave runner, Fruit Ninja
	Full Class Debrief: students reflected by describing their experiences in relation to the guiding questions
Months 2-4: first full-time clinical education experience I	Students completed an 8-week summer clinical experience between semesters 2 and 3. Appropriate settings for this first clinical experience include: outpatient musculoskeletal and neurorehabilitation settings, subacute and acute rehabilitation facilities.
Months 4-8: students begin semester 3 of the curriculum (Fall)	In tutorials, labs, and large group discussions, students discuss patients with disorders or dysfunction of the cardiopulmonary, integumentary, and complex multi-system problems. Students apply that knowledge to more advanced problem-solving and patient management from examination through evaluation, diagnosis, prognosis, and intervention.
Month 8: semester 3; lab activity #2; 45 minutes with small groups in each station followed by a 30-minute debrief (Supplement 4)	^a)^Station 1: 20-yr male; s/p spinal cord injury
	• Stability: seated postural control
	• AVG system: Nintendo Wii
	• Game(s): Mario Cart, Cow Run
	^b)^Station 2: 56-yr male; s/p motor vehicle accident with complex medical diagnoses
	• Mobility: using a rolling a walker
	• AVG system: XBox One Kinect
	• Game(s): soccer, rock climbing, wave runner, Fruit Ninja, bowling
	^c)^Station 3: 10-yr female with asthma
	• Mobility Plus: aerobic exercise
	• AVG system: Nintendo Switch
	• Game(s): Just Dance
	Full-class debrief: students reflected by describing their experiences in relationship to the guiding questions
Post-lab survey	Post-test survey was emailed and completed after lab activity #2

s/p, status post; AVG, active video game.

a) Stability: maintain a posture or orientation of the trunk and limbs to: allow movement of other body segments, hold body and body segments in a required game position, and resist perturbations.

b) Mobility: movement of body segments to reach a target, avoid obstacles, assume required positions, or “drive” or “steer” the game task.

c) Mobility Plus: a higher-level movement required, including more athletic balance or mobility tasks such as jumping, lunging, or running in place.

^a-c)^Defined by Levac et al. [11].

**Table 2.** Demographic information

Characteristic	Cohort 2017 (n=60)	Cohort 2018 (n=55)
Age (yr)	22.0±0.84 (21.0–29.0)	22.0±0.94 (21.0–26.0)
Undergraduate degree		
Exercise science	37 (61.7)	46 (54.2)
Athletic training	7 (11.7)	5 (9.1)
Biology	4 (6.7)	2 (3.6)
Psychology	5 (8.3)	1 (1.8)
Kinesiology	2 (3.3)	1 (1.8)
Health science	1 (1.7)	0
3+3 physical therapy	3 (5.0)	0
Nutritional sciences	1 (1.7)	0
Gender		
Female	39 (65.0)	40 (72.7)
Male	21 (35.0)	15 (27.3)
Confidence operating Xbox (%)	49.5±32.1	
Confidence operating Wii (%)	70.4±27.0	
Confidence using for physical therapy intervention (%)	55.1±25.4	

Values are presented as mean±SD (range), number (%), or mean±SD, unless otherwise stated. SD, standard deviation.

**Table 3.** Changes in students’ perceived confidence pre- and post-intervention, where the maximum score was 100

Variable	Cohort 1		Cohort 2	
	Pre-intervention	Post-intervention	Pre-intervention	Post-intervention
General use of gaming	65.0 (51.5–74.5)	86.0 (78.0–94.0)	70.0 (53.0–80.0)	90.0 (84.0–96.0)
Game selection	66.0 (51.0–75.0)	86.0 (78.0–91.0)	67.0 (51.0–76.0)	90.0 (83.0–94.0)
Plan of care	51.0 (40.0–60.0)	74.0 (64.0–80.0)	58.0 (44.0–70.0)	88.0 (80.0–94.0)
Set-up	60.0 (43.0–64.0)	77.0 (70.0–81.0)	67.0 (50.0–80.0)	93.0 (87.0–100.0)
Documentation	53.0 (40.0–60.0)	77.0 (70.0–81.0)	58.0 (47.0–72.5)	83.0 (80.0–93.0)
Setting	45.0 (33.5–56.0)	70.0 (61.5–80.0)	56.0 (40.5–66.0)	84.0 (78.0–92.0)
Total	57.0 (44.0–63.5)	79.0 (73.0–85.0)	61.0 (48.0–71.0)	89.0 (80.0–93.0)

Values are presented as median (interquartile range).

## Footnotes

**Data availability**
Data files are available from Harvard Dataverse: https://doi.org/10.7910/DVN/AUYSTE Dataset 1. Pre-post cohort 1 2017.

Dataset 2. Pre-post cohort 2 2018.

Dataset 3. Differences in baseline confidence cohort 1 and cohort 2.

## Supplementary materials

Data files are available from Harvard Dataverse: https://doi.org/10.7910/DVN/AUYSTE.

Supplement 1. Tutorial case: Matthew Mills, an individual with a mild acquired brain injury.

Supplement 2. Semester 2: videogaming lab activity #1.

Supplement 3. Video file: Doctor of Physical Therapy students participating in active video gaming during lab experience.

Supplement 4. Semester 3: videogaming lab activity #2.

Supplement 5. Student survey of perceived confidence with video gaming as a physical therapy intervention.

Supplement 6. Audio recording of the abstract.
