# Supplementary material for: Doctoral physical therapy students’ increased confidence following exploration of active video gaming systems in a problem-based learning curriculum in the United States: a pre- and post-intervention study
Source: J Educ Eval Health Prof. 2022 Apr 26;19:7. doi: 10.3352/jeehp.2022.19.7 (PMC9247715; doi:10.3352/jeehp.2022.19.7)
Supplement: Supplementary file 4 — Supplement 1. Tutorial case: Matthew Mills, an individual with a mild acquired brain injury. [file jeehp-19-07-suppl1.docx]

**Supplement 1.** Tutorial case: Matthew Mills, an individual with a mild acquired brain injury

Matthew Mills is a 27-year-old avid snowboarder who sustained a concussion in a collision with another snowboarder 3 weeks ago. He was momentarily unconscious, but responsive to voice and pain stimuli at the time of accident. On arrival at the emergency room, he had a Glasgow coma score of 13, a Mini-Mental State Exam score of 24 (he had difficulty with short term memory, serial-7 subtraction task, and the figure copying task) but no focal neurological symptoms. Magnetic resonance imaging was essentially normal, with no signs of subarachnoid, epidural or subdural hematoma, or of significant contusion or hemorrhage in the telencephalon or brainstem. Admitted for overnight observation, he was discharged to home with his fiancé.

He stayed home from his job as an elementary school physical education teacher for the next week, complaining of headaches, distractibility, difficulty sustaining attention/vigilance to tasks and dividing attention for dual-tasking, more disorganized picking up all necessary items at the grocery store, and difficulty following the action while watching his favorite sports teams on TV. He returned to his job last week, but has been having significant difficulty with “visual information processing,” making frequent errors in judging speed and distance in activities with catching/throwing components, and having to “work harder than normal” when trying to read while studying for his graduate class. He also reports that he is having difficulty finding utensils and tools in the disorganized drawers of his desk and kitchen, and has to concentrate to avoid spilling when pouring his coffee into his favorite mug in the morning. He states that “he suddenly has a sense of what it means to have a learning disability.” He is afraid that he will not be able to return to the visual-processing demands of snowboarding and is wondering if he went back to work too quickly. Matt began reading up on concussion and came across information on the risk of second impact syndrome and made a note to ask his PT about it. While reading more about his injury, he also came across information supporting baseline testing in student athletes at risk for concussion. This got him reflecting on past students who have returned to school after having had a mild traumatic brain injury (MTBI) and wondering if their performance in school was subsequently affected in the short and/or long term.

Matt’s neurologist refers him to an ophthalmologist/neuropsychologist team for further assessment. In the initial consultation, the MD and PhD tell Matt that they will examine his visual perceptual performance in 3 domains: visual-spatial skills, visual analysis skills, and visual motor skills. They explain that it is not uncommon to experience visual perceptual impairment (visual information processing impairment) after MTBI. They suggest that, given that his impairments are impacting on his function at work and in his leisure activities, they will likely refer him to an occupational or physical therapist for “rehabilitation,” vision therapy, and possibly speech-language pathologist for strategies to succeed at work.

**Guiding questions:**

• What areas of the brain are involved in visual perception? Is there a difference in L and R hemispheres?

• How does visual-perception contribute to/influence gross and skilled movement during functional tasks?

• What are the immediate and longer-term effects of mild head injury on brain function?

• In what compartments of the brain and meninges might bleeding occur? How are signs/symptoms of each differentiated? How is each managed?

• What might a visual processing information deficit be confused with/mistaken for?

• What is the prognosis post-concussion? Is there a difference between children vs. adults?

• How are visual processing impairments treated?

• What guides determination of a safe time frame for return to play and work? Is there a different approach in children vs. adults?

• What evidence exists in the literature regarding the use of XBox Kinect and other videogaming consoles in rehabilitation?
