# Supplementary material for: Doctoral physical therapy students’ increased confidence following exploration of active video gaming systems in a problem-based learning curriculum in the United States: a pre- and post-intervention study
Source: J Educ Eval Health Prof. 2022 Apr 26;19:7. doi: 10.3352/jeehp.2022.19.7 (PMC9247715; doi:10.3352/jeehp.2022.19.7)
Supplement: Supplementary file 5 — Supplement 2. Semester 2: videogaming lab activity #1. [file jeehp-19-07-suppl2.docx]

**Supplement 2.** Semester 2: videogaming lab activity #1

Students will explore the use of video gaming in order to select games and develop plan of care for patients listed below:

**Stability (working on stability without an AD)**

Fong is a 73-year-old female s/p L MCA stroke 1.5 months ago. Amb 100 feet with a hemiwalker ModI with an articulating AFO. Transfers sit to stand with supervision from EOB and min (A) from low couch. Postural control: Static sitting (I) on mat in midline w/o UE support ×3 minutes. Standing (I) with UE support from L HW and weight shifted toward L ×2 minutes. Reluctant to stand without support of HW, but able to do so with (S) ×1 minute and Borg RPE of 6/10. Anticipatory: Sitting (I) to reach on a diagonal towards L side and forward. (S) to weight shift across midline and reach on a diagonal towards R side at least 50% of “typical” excursion. Standing (I) to weight shift and reach towards L however self-limits sway envelope by 50%; min (A) to reach diagonally or laterally toward R in standing, although reluctant to do so and requires facilitation at R UE. Reactionary: Sitting and standing- Appropriate balance strategies (equilibrium responses) observed following min to mod A/P, L lateral, and L diagonal perturbations. Delayed and ineffective step and reach strategies (protective responses), R extremities >L extremities, observed in response to min R lateral and R diagonal perturbations, requiring mod A to prevent LOB.

**Mobility (she likes dancing, poor dynamic postural control)**

Jane Righetti is an 82-year-old woman with Parkinson’s disease (Hoehn & Yahr stage 3), diagnosed 12 years ago and pharmacologically managed with Sinemet, with the recent addition of Neupro. Jane was an avid ballroom dancer until her husband’s death last year, and continues to be a music lover. Ambulates 200 ft with forward flexed posture at trunk, hips, knees, dec step length bilaterally with shuffling gait pattern and lacks hip extension at terminal stance. No assistive device. Strength: 3+/4- out of 5 BUE and BLE, but is slow to initiate movement and lacks active end range in most directions of trunk as well as extremities.

Sit to stand: modified independent, increase time and difficulty initiating movement. Reactionary postural control requires max A to recover from backwards perturbation. Outcome measures: Four square step test: 22.2 seconds, timed up and go: 42 seconds, difficulty getting up from chair and turning. TUG-cog: 63 seconds, functional reach: 2 inches. On second trial, pt lost balance with multiple shuffling steps in forward direction and needed maximum assistance to recover.

**Mobility Plus** **(high level with visual processing issues–a snowboarder, young)**

Matthew Mills is a 27-year-old, avid snowboarder, who sustained a concussion in a collision with another snowboarder 3 weeks ago. He returned to his job last week, but has been having significant difficulty with “visual information processing,” making frequent errors in judging speed and distance in activities with catching/throwing components, and having to “work harder than normal” when trying to read while studying for his graduate class. He also reports that he is having difficulty finding utensils and tools in the disorganized drawers of his desk and kitchen, and has to concentrate to avoid spilling when pouring his coffee into his favorite mug in the morning. He is afraid that he will not be able to return to the visual-processing demands of snowboarding and is wondering if he went back to work too quickly.

By the end of this lab, students will answer the following questions:

1. What game/games would you select to play with the patients? And why?

2. What impairments and activities can you address using the video games you played with today? Link game with impairment and/or activity

3. How would you create a plan of care and select a game that would maximize success and minimize frustration?

4. How would you guard and/or use assistive devices while gaming to maximize the patient’s safety?

5. How would you prescribe the treatment using gaming? Consider FITT (Frequency, Intensity, Type and Timing)

6. How would you monitor your patient?

7. What assessments would you use to determine if the intervention worked?
