# Supplementary material for: Doctoral physical therapy students’ increased confidence following exploration of active video gaming systems in a problem-based learning curriculum in the United States: a pre- and post-intervention study
Source: J Educ Eval Health Prof. 2022 Apr 26;19:7. doi: 10.3352/jeehp.2022.19.7 (PMC9247715; doi:10.3352/jeehp.2022.19.7)
Supplement: Supplementary file 7 — Supplement 4. Semester 3: videogaming lab activity #2. [file jeehp-19-07-suppl3.docx]

**Supplement 4.** Semester 3: videogaming lab activity #2

Explore video-gaming in order to select appropriate games to include in your POC for the following patients (consider how to address specific impairments and activity limitations):

**Stability (impaired static and dynamic postural control)–Xbox**

Lu-Chen is a 20 y/o male with a C6 AIS A SCI s/p GSW being seen in your outpatient clinic after 8 weeks of inpatient and 2 weeks of home care services. He just had his halo removed last week. On eval, he is (I) to maintain static short sitting balance with (B) UE support ×2 minutes and is SBA with 1 UE support ×30 seconds. He has poor ability to tolerate internal perturbations such as head turns or reaching as observed by falling in that direction. After set-up, he is CG A for SB transfer to even surfaces. Lu-Chen wants to work towards maintaining his balance for longer periods of time with 1 to no UE support, to turn his head w/o falling over, to reach more confidently within his base of support, and to “have some fun in the process.”

**Mobility (impaired dynamic postural control and mobility)–Xbox One Kinect**

Marvin is a 56 y/o male severely injured in an MVA 9 weeks ago. He had a 5 week stay in acute care to manage a complicated medical course including a pneumothorax, chest tubes, blunt chest trauma, temporary pacemaker secondary to 1st degree AV block, an intracranial bleed with craniotomy and ICP bolt, and a left standard length transfemoral amputation. PmHx includes Type 2 DM, Non-STEMI ×2 years ago, venous insufficiency, and HTN. He has been your patient in acute rehab over the last 4 weeks for pre-prosthetic training and is (I) with bed mobility, (S) transferring with RW, close (S) amb ×150 with RW, and close (S) stepping up a 4” step with RW. His prosthetic was delivered and his stay has been justified to prepare him for walking short distances at home. Your goals are to work with him on weight acceptance, single limb support, and reaching minimally outside of his base of support.

**Mobility Plus (high level with impaired aerobic capacity)–Nintendo Wii Switch**

Brenda is a 10 y/o female s/p status asthmaticus 1 week ago requiring emergency medical care. PMH includes multiple admissions for status asthmaticus. Her mother and teachers are concerned about her ability to participate in physical education classes and get to/from her 3rd floor classroom via the stairs due to her lack of endurance. They also worry that she has been reluctant to play with her peers due to fear of exercise triggering her asthma. She does, however, love to watch her older sister dance and play sports but has not wanted to sign up for classes herself. After a parent-teacher meeting, Brenda is referred to school-based physical therapy to address her impairments. She is (I) with all functional mobility, reported 3/10 SOB on an informal assessment of aerobic capacity performed via brisk walk from the front door of the school to her classroom on the 3rd floor, and was only able to complete 4 laps during a recent 20-meter PACER test (Progressive Aerobic Cardiovascular Endurance Run or Beep test) administered in gym class due to RR of 30, HR of 120, and 6/10 on BORG RPE and dyspnea scales (low end norm for girls age 10=7 laps).

By the end of this lab please answer the following questions:

1. What game/games would you select to play with the patients? And why?

2. What impairments and activities can you address using the video games you played with today? (Link specific game(s) with impairment and/or activity)

3. How would you create a plan of care and select a game that would maximize success and minimize frustration?

4. How would you guard and/or use assistive devices while gaming to maximize each patient’s safety?

5. How would you prescribe the treatment using gaming? Consider FITT (Frequency, Intensity, Type, and Timing)

6. How would you monitor your patient?

7. What assessments would you use to determine if the intervention worked?
