# Supplementary material for: Doctoral physical therapy students’ increased confidence following exploration of active video gaming systems in a problem-based learning curriculum in the United States: a pre- and post-intervention study
Source: J Educ Eval Health Prof. 2022 Apr 26;19:7. doi: 10.3352/jeehp.2022.19.7 (PMC9247715; doi:10.3352/jeehp.2022.19.7)
Supplement: Supplementary file 8 — Supplement 5. Student survey of perceived confidence with video gaming as a physical therapy intervention. [file jeehp-19-07-suppl4.docx]

**Supplement 5.** Student survey of perceived confidence with video gaming as a physical therapy intervention

**General use of gaming**

This scale is used to measure your confidence using video games to supplement your physical therapy treatment. For each of the following questions, please rate how confident you are performing each activity:

| 0 | 10 | 20 | 30 | 40 | 50 | 60 | 70 | 80 | 90 | 100 |
| --- | --- | --- | --- | --- | --- | --- | --- | --- | --- | --- |
| No confidence | | Limited confidence | | | Moderate confidence | | | High confidence | | |

1. Operate an Xbox Kinect video game console

2. Operate a Nintendo Wii video game console

3. Use video games as a physical therapy treatment with patients

4. Recognize that a patient may benefit from the use of video games as a physical therapy intervention

5. Educate a patient on the benefits of video games in physical therapy practice

**Game selection**

This scale is used to measure your confidence using video games to supplement your physical therapy treatment. For each of the following questions, please rate how confident you are performing each activity:

| 0 | 10 | 20 | 30 | 40 | 50 | 60 | 70 | 80 | 90 | 100 |
| --- | --- | --- | --- | --- | --- | --- | --- | --- | --- | --- |
| No confidence | | Limited confidence | | | Moderate confidence | | | High confidence | | |

6. Prioritize the patient’s body structure/function impairments, per the ICF model to be addressed.

7. Choose a game to address the prioritized body structure/function impairment(s) the patient is having difficulty with

8 Prioritize the patient’s activity limitations, per the ICF model to be addressed.

9. Choose a game to address the prioritized activity limitation(s) the patient is having difficulty with

10. Select a game that minimizes frustration and maximizes patient success

11. Select a game to improve the patient’s stability

12. Select a game to improve the patient’s mobility

**Plan of care**

This scale is used to measure your confidence using video games to supplement your physical therapy treatment. For each of the following questions, please rate how confident you are performing each activity:

| 0 | 10 | 20 | 30 | 40 | 50 | 60 | 70 | 80 | 90 | 100 |
| --- | --- | --- | --- | --- | --- | --- | --- | --- | --- | --- |
| No confidence | | Limited confidence | | | Moderate confidence | | | High confidence | | |

13. Determining frequency

14. Determining intensity

15. Determining time

16. Determining type of exercise

17. Prescribing a home exercise program

**Set up**

This scale is used to measure your confidence using video games to supplement your physical therapy treatment. For each of the following questions, please rate how confident you are performing each activity:

| 0 | 10 | 20 | 30 | 40 | 50 | 60 | 70 | 80 | 90 | 100 |
| --- | --- | --- | --- | --- | --- | --- | --- | --- | --- | --- |
| No confidence | | Limited confidence | | | Moderate confidence | | | High confidence | | |

18. Safely set up a patient to use the equipment

19. Safely guard a patient while using the equipment

20. Identify when more than one person is needed to safely perform the intervention

**Documentation**

This scale is used to measure your confidence using video games to supplement your physical therapy treatment. For each of the following questions, please rate how confident you are performing each activity:

| 0 | 10 | 20 | 30 | 40 | 50 | 60 | 70 | 80 | 90 | 100 |
| --- | --- | --- | --- | --- | --- | --- | --- | --- | --- | --- |
| No confidence | | Limited confidence | | | Moderate confidence | | | High confidence | | |

21. The use of the video game as part of the “objective” in a SOAP note

22. The patient’s response to the intervention as part of the “assessment” in a SOAP note

23. The “plan” for progressing a patient using videogames as an intervention in a SOAP note

**Setting**

This scale is used to measure your confidence using video games to supplement your physical therapy treatment. For each of the following questions, please rate how confident you are in using video games in each setting:

| 0 | 10 | 20 | 30 | 40 | 50 | 60 | 70 | 80 | 90 | 100 |
| --- | --- | --- | --- | --- | --- | --- | --- | --- | --- | --- |
| No confidence | | Limited confidence | | | Moderate confidence | | | High confidence | | |

24. Acute care

25. Acute rehab

26. Long-term acute rehab (LTAC)

27. Subacute rehab (skilled nursing facility)

28. Outpatient

**Demographics**

29. Gender: male/female

30. Age (drop down)

31. Undergraduate degree: Exercise Science, Psychology, Biology, Athletic Training, Other: Please Specify
